# The *Tetrahymena* metallothionein gene family: twenty-one new cDNAs, molecular characterization, phylogenetic study and comparative analysis of the gene expression under different abiotic stressors

**DOI:** 10.1186/s12864-016-2658-6

**Published:** 2016-05-10

**Authors:** Patricia de Francisco, Laura María Melgar, Silvia Díaz, Ana Martín-González, Juan Carlos Gutiérrez

**Affiliations:** Departamento Microbiología-III, Facultad de Biología. C/José Antonio Novais, 12, Universidad Complutense de Madrid (UCM), 28040 Madrid, Spain; Universidad Castilla-La Mancha, Campus Tecnológico de la fábrica de armas, Edificio Sabatini. Av. Carlos III, s/n. 45071 Toledo, Spain

**Keywords:** *Tetrahymena* metallothioneins, cDNAs, Structural characterization, Gene duplication, Phylogeny, Gene expression

## Abstract

**Background:**

Ciliate metallothioneins (MTs) are included in family 7 of the MT superfamily. This family has been divided into two main subfamilies: 7a or CdMTs and 7b or CuMTs. All ciliate MTs reported have been isolated from different *Tetrahymena* species and present unique features with regard to standard MTs. Likewise, an expression analysis has been carried out on some of MT genes under metal stress, corroborating their classification into two subfamilies.

**Results:**

We isolated 21 new cDNAs from different *Tetrahymena* species to obtain a wider view of the biodiversity of these conserved genes. Structural analysis (cysteine patterns) and an updated phylogenetic study both corroborated the previous classification into two subfamilies. A new CuMT from a *Tetrahymena*-related species *Ichthyophthirius multifiliis* was also included in this general analysis. We detected a certain tendency towards the presentation of a CdMT tri-modular structure in *Borealis* group species with respect to *Australis* group. We report for the first time a semi-complete paralog duplication of a CdMT gene originating a new CdMT gene isoform in *T. malaccensis*. An asymmetry of the codon usage for glutamine residues was detected between Cd- and CuMTs, and the phylogenetic implications are discussed. A comparative gene expression analysis of several MT genes by qRT-PCR revealed differential behavior among them under different abiotic stressors in the same *Tetrahymena* species.

**Conclusions:**

The *Tetrahymena* metallothionein family represents a quite conserved protein structure group with unique features with respect to standard MTs. Both Cd- and CuMT subfamilies present very defined and differentiated characteristics at several levels: cysteine patterns, modular structure, glutamine codon usage and gene expression under metal stress, among others. Gene duplication through evolution seems to be the major genetic mechanism for creating new MT gene isoforms and increasing their functional diversity.

**Electronic supplementary material:**

The online version of this article (doi:10.1186/s12864-016-2658-6) contains supplementary material, which is available to authorized users.

## Background

Metallothioneins (MTs) constitute a superfamily of small ubiquitous cytosolic proteins (25–82 aa, 2.5–8.0 KDa) which are able to bind metal cations through their numerous cysteine (Cys) residues (18–23 Cys organized in conserved domains). Several functions have been proposed for these proteins [1], such as protection from toxic metals and oxidative stress [2], essential-metal homeostasis [3] and protection against xenobiotics [4]. Likewise, they have been implicated in protection against neurodegenerative diseases [5], apoptosis and the biology of aging [6], as well as in processes of development and cellular differentiation [7]. This is the reason why these proteins are considered to be multifunctional proteins, involved in many diverse cellular processes.

Since 1957, when the first MT was described [8], more than 20,000 articles have been dedicated to the study of the structure, biochemical or functional features and gene expression analysis of MTs [9]. MTs have been reported in both eukaryotic organisms (protists, yeasts, phylamentous fungi, higher plants and animals) and prokaryotic cells (cyanobacteria, γ- and α-Proteobacteria and some Firmicutes) [10, 11]. To date, the presence of MTs has been reported among ciliated protozoa in only two genera (*Tetrahymena* and *Paramecium*) [12–17]. However, with regard to the putative *Paramecium* MT gene, no experimental data has been reported on its gene expression under metal stress [17]. Experiments on expression are therefore needed in order for it to be considered as a real MT. Attempts to classify MTs have been made by different authors. In 1999, Binz and Kägi [18] proposed a classification based on 15 families, defining one MT family for each main taxonomic group of organisms except fungi, to give six different sets. In this classification ciliate MTs are included in family 7, which has been subsequently divided into two main subfamilies: 7a or cadmium-binding MTs (CdMTs) and 7b or copper-binding-MTs (CuMTs) [19]. At present, subfamily 7a includes 14 CdMTs from different *Tetrahymena* species: two from *T. pyriformis* (TpyrMT-1 and TpyrMT-2) [14, 20], three from *T. thermophila* (TtheMTT1, TtheMTT3 and TtheMTT5) [19, 21], one from *T. tropicalis* (TtroMTT1) [22], one from *T. rostrata* (TrosMTT1) [23], one from *T. pigmentosa* (TpigMT-1) [15], one from *T. vorax* (TvorMT1), one from *T. mobilis* (TmobMT1) and four from *T. hegewischi* (ThegMT1, ThegMT2, ThegMT3 and ThegMT4) [16]. Subfamily 7b contains 7 CuMTs: two from *T. thermophila* (TtheMTT2 and TtheMTT4) [19, 24], one from *T. pigmentosa* (TpigMT-2) [15], two from *T. tropicalis* (TtropMT1 and TtropMT2), one from *T. rostrata* (TrosMTT2) [23] and one from an unspecified *Tetrahymena* sp1.7 (sp1.7-MT1). The two subfamilies differ mainly in their typical Cys residue clustering [12, 13].

*Tetrahymena* MTs have unique features with respect to MTs from other organisms. Their lengths (78–191 aa) and molecular masses (8.2–20 KDa) are higher than MTs from vertebrates (25–82 aa; 2.5–8.0 KDa); therefore, they have a considerably higher number of Cys residues and a potentially larger metal binding capacity per MT molecule [12, 13, 25]. As detected in other organisms, a remarkably regular and hierarchical modular structural organization has been observed in *Tetrahymena* MTs, mainly CdMTs [12, 13, 16, 19]. Several authors have proposed evolutionary history models for these MT genes, based on their modular and submodular structure and gene duplication as the main mechanism involved in MT evolution [12, 16, 19, 26, 27]. In general, MT genes can be induced by an extensive range of different environmental biotic or abiotic stressors, such as metal(loid)s, oxidative agents, heat or cold shocks, hormones, cytokines, pH changes, starvation, and a large variety of organic chemicals or drugs. Likewise, *Tetrahymena* MT gene expression can be induced by very diverse stressors [12, 13, 19, 20, 23, 24].

In this study we report the cloning and characterization of 21 new MT genes (cDNAs) isolated from five *Tetrahymena* species never before analysed: *T. borealis*, *T.elliotti*, *T.americanis*, *T. patula* and *T. malaccensis*, selected from the two main taxonomic *Tetrahymena* groups (*australis* and *borealis*) [28]. Thereby increasing the CdMT sequences by about 46 % and the CuMT sequences ~ 56 % in the *Tetrahymena* MT family, to give us a broader view of these highly conserved molecules. Furthermore, a new putative CuMT from the *Tetrahymena* related species and fish parasite *Ichthyophthirius multifiliis* has been incorporated into the actual *in silico* analysis of the MT sequence list (after exploration of its already sequenced macronuclear genome). Owing to the high nucleotide sequence identities among these MT genes, it has only been possible to analyze six of them by quantitative RT-PCR under different metal(loid)s and other environmental stressors.

## Results and discussion

### New Cd- and CuMTs from different *Tetrahymena* species

At present, there are about 42 known species of the genus *Tetrahymena*. These have been classified into two main groups: *australi*s and *borealis*, according to the small subunit ribosomal gene (SSrRNA, 17-18S) [28]. At the same time, the *borealis* group is divided into three groups or ribosets: RSA1, RSA2 and RSB. In order to isolate new MT genes, *Tetrahymena* species from the SSrRNA phylogenetic tree were selected [28] to obtain a broader wide view of the biodiversity of these conserved genes. Table 1, shows all the *Tetrahymena* MTs reported up to now, including the new MTs isolated in this study. So far, 13 *Tetrahymena* species have been analyzed (~31 % of the total known species): 9 from the *Borealis* group and 4 from the *Australis* group. From these, a total of 26 CdMT and 15 CuMT sequences have been reported (Table 1). In the present study, we incorporated 12 new CdMT cDNA sequences (~46 % more) and 9 new CuMT cDNA sequences (~56 % more), so thereby considerably increasing our knowledge of these conserved genes. Also included in this analysis was a new CuMT sequence from the ciliate parasite (that causes white spot disease in fresh water fishes) *Ichthyophthirius multifiliis* (taxonomically related to *Tetrahymena*, because both are located in the same *Hymenostomatia* subclass), giving a total of 16 CuMT sequences considered in this study (Table 1). Of the new CdMT cDNA sequences, one (*TmalaMTT1*) is identical to the previously reported *TmalMT1*, also isolated from the *T.malaccensis* [16]. However, in this putative cDNA [GenBank: HQ166894] 5’or 3’UTRs have not been reported, while our *TmalaMTT1* cDNA already presents both UTR regions [GenBank: KU167646].

**Table 1:** Present ciliate metallothioneins

Taxonomic group	Ciliate species	CdMT	CuMT	Total
*Borealis* (RSA2)	*T. borealis*	TborMTT1^a^	TborMTT3^a^	7
		TborMTT2^a^	TborMTT4^a^	
			TborMTT6^a^	
			TborMTT7^a^	
			TborMTT8^a^	
*Borealis* (RSA2)	*T. elliotti*	TelliMTT1^a^	TelliMTT6^a^	4
		TelliMTT2^a^	TelliMTT8^a^	
*Borealis* (RSA1)	*T. malaccensis*	TmalaMTT1^a^	TmalaMTT5^a^	5
		TmalaMTT2^a^		
		TmalaMTT3^a^		
		TmalaMTT4^a^		
*Borealis* (RSA2)	*T. mobilis*	TmobMT1	?	1
*Borealis* (RSB)	*T. pyriformis*	TpyrMT-1	?	2
		TpyrMT-2		
*Borealis* (RSA2)	*T. rostrata*	TrosMTT1	TrosMTT2	2
*Borealis* (RSA1)	*T. thermophila*	TtheMTT1	TtheMTT2	5
		TtheMTT3	TtheMTT4	
		TtheMTT5		
*Borealis* (RSA2)	*T. tropicalis*	TtroMTT1	TtropMT1	3
			TtropMT2	
*Borealis* (RSB)	*T. vorax*	TvorMT1	?	1
*Australis* (RSC)	*T. americanis*	TamerMTT1^a^	TamerMTT3^a^	3
		TamerMTT2^a^		
*Australis* (RSC)	*T. hegewischi*	ThegMT-1	?	4
		ThegMT-2		
		ThegMT-3		
		ThegMT-4		
*Australis* (RSC)	*T. patula*	TpatMTT1^a^	?	2
		TpatMTT2^a^		
*Australis* (RSC)	*T. pigmentosa*	TpigMT-1	TpigMT-2	2
*Ichthyophthiriidae*	*I. multifiliis*	?	ImMTT2^a^	1
Total	14	26	16	42

^a^New Cd- or CuMTs reported in this study. RS: riboset. ? : unknown

**Fig. 1:**
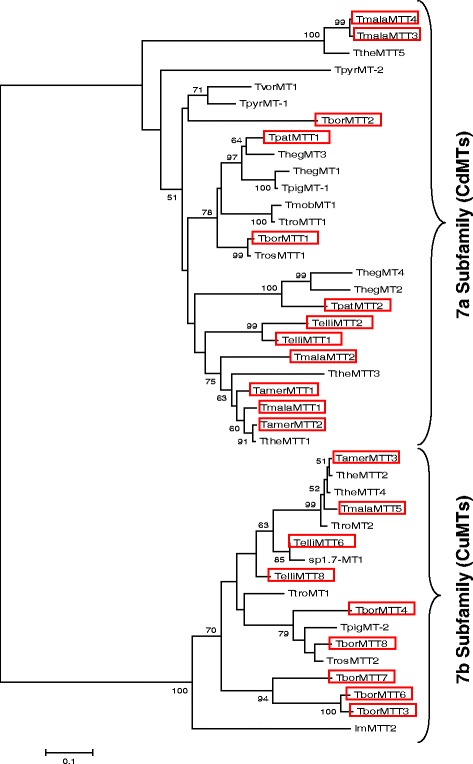
Phylogenetic tree of all ciliate metallothioneins. Two well defined groups corresponding to both MT subfamilies are observed. Multiple alignments of amino acid sequences were obtained by using the T-Coffee program. Numbers indicate bootstrap values (<50 % are not shown) from 2000 replicates. Brach lengths are drawn to scale as indicated by the scale bar. The names of the new MT sequences reported in this paper are indicated into the red boxes. See Table 1 for ciliate species identification

**Fig. 2:**
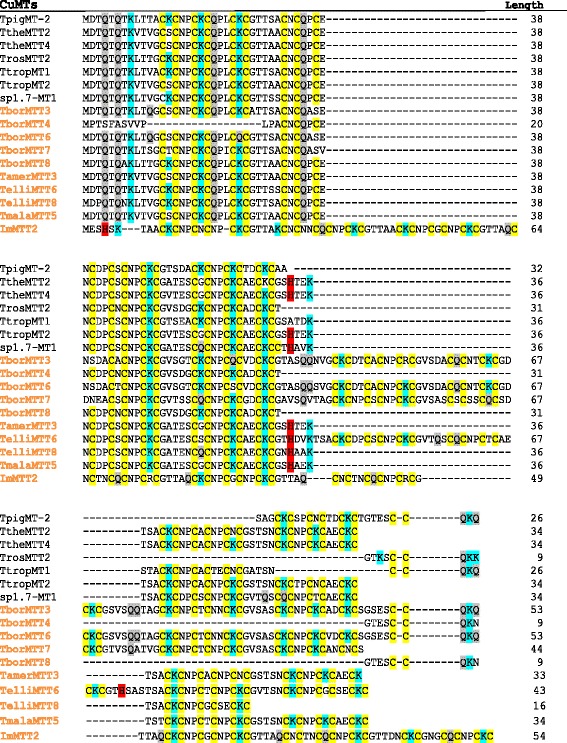
Molecular structure of subfamily 7b (CuMTs). Multiple alignments were made using the T-Coffee program, followed by visual inspection and manual adjustment. The new CuMT sequences reported in this paper are indicated with colored (orange) names. Yellow shaded regions indicate conserved Cys (C) residues. Blue and grey shaded regions indicate Lys (K) or Gln (Q) residues, respectively. His (H) residues are indicated by red shading. See the text for further explanation

**Fig. 3:**
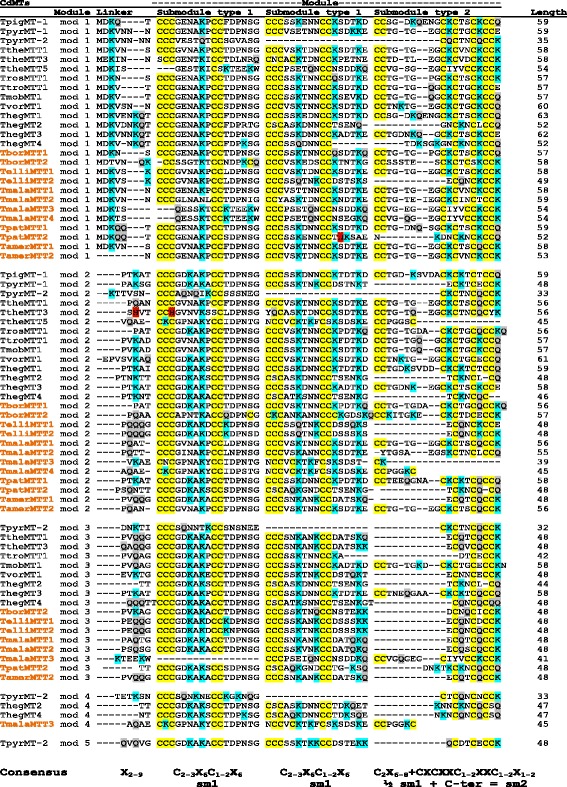
Modular structure of subfamily 7a (CdMTs). Multiple alignments were made using the T-Coffee program, followed by visual inspection and manual adjustment. The new CdMT sequences reported in this paper are indicated with colored (orange) names. Yellow shaded regions indicate conserved Cys (C) residues. Blue and grey shaded regions indicate Lys (K) or Gln (Q) residues, respectively. His (H) residues are indicated by red shading. sm1: submodule-1, sm2: submodule-2. See the text for further explanation

Ciliate MTs (family 7) [18] have been divided into two subfamilies: 7a (CdMTs) and 7b (CuMTs) [19]. An updated phylogenetic tree drawn up to include the new inferred amino acid sequences from all the new ciliate MT genes (Fig. 1), confirms the previous classification into two large groups or subfamilies: CdMTs and CuMTs. This is due to the strict Cys residue patterns or modular configuration of these proteins in both MT groups (Figs. 2 and 3). Although the CuMT sequence from *I. multifiliis* (ImMTT2) is clearly separated from the rest of the *Tetrahymena* CuMTs, it is completely integrated in the CuMT group or subfamily (Fig. 1). In general, MTs from the same *Tetrahymena* species are located close together in the tree, although this is not always the case. This may indicate a certain convergent evolution of these genes into the *Tetrahymena* genus, or a common ancestor gene structure which originated very similar proteins through the different *Tetrahymena* species.

### Cysteine patterns

Cysteine (Cys) residues are the main amino acids in MT composition because they are the chelating points for metals, forming metal-thiolate clusters [29, 30]. The total amount of *Tetrahymena* MT Cys residues is considerable (up to 54 residues for CdMTs) (Table 2), due to the longer length of these molecules with respect to standard MTs. However, the average Cys percentage of these MTs lies within the average Cys percentage values for standard MTs (7–21 Cys residues) (Table 2). Traditional Cys (C) clusters appear in *Tetrahymena* CdMTs as in standard MTs, such as: XCCX (34.94 %), CXC (10.72 %) and XXCXX or non-clustered Cys (4.16 %) (Table 2). In addition, they have other Cys clusters that are almost exclusive to *Tetrahymena* CdMTs: CCC (32.44 %), CXCC (16.91 %) or CXCXC (0.83 %) (where “X” is any other amino acid different to Cys) (Table 2). These unusual Cys clusters also appear in the MTs of certain other organisms, such as the annelid *Eisenia foetida* (CCC) [GenBank: P81695]*,* the yeast *Yarrowia lipolytica* (CCC) [GenBank: Q9HFD0], the arthropod *Callinectes sapidus* (CCC and CXCC) [GenBank: AAF08966]*,* the mollusk *Crassostrea virginica* (CCC and CXCC) [GenBank: AAZ94898]*,* the American lobster *Homarus americanus*(CXCC) [GenBank: P29499], the amphibious *Xenopus laevis*(CXCC) [GenBank: AAB60616]*,* the yeast *Saccharomyces cerevisiae* (CXCXC) [GenBank: AAA66061], the nematode *Caenorhabditis elegans* (CXCXC) [GenBank: P17511] and the purple sea urchin *Strogylocentrotus purpuratus* (CXCC) [GenBank: P04734]. In *Tetrahymena* CdMTs, CCC and XCCX are the most abundant clusters (Table 2). However, in ciliate CuMTs CCC, CXCC and CXCXC clusters are almost absent (Table 2). On the other hand, the predominant cluster in these CuMTs is CXC (92.34 %), as in MTs from other organisms.

**Table 2:** Distribution of Cys clusters among *Tetrahymena* MTs

CdMT	CCC	CXCC	CXCXC	XCCX	CXC	XXCXX	Total Cys	% Cys^(a)^	Length
TpigMT-1	4	2	0	6	2	0	34	28.81	118
TpyrMT-1	4	2	0	5	1	1	31	28.97	107
TpyrMT-2	6	5	0	6	4	1	54	29.83	181
TtheMTT1	6	3	0	8	2	1	48	29.63	162
TtheMTT3	2	2	1	9	3	3	42	25.93	162
TtheMTT5	1	1	0	5	1	6	24	24.24	99
TrosMTT1	4	2	0	6	2	0	34	30.09	113
TtroMTT1	6	3	0	8	2	0	47	30.13	156
TmobMT1	6	3	0	9	3	0	51	29.65	172
TvorMT1	6	3	0	8	2	1	48	28.40	169
ThegMT1	4	2	0	6	2	0	34	27.87	122
ThegMT2	4	1	0	8	7	4	49	25.65	191
ThegMT3	6	3	0	9	3	0	51	28.65	178
ThegMT4	5	1	0	8	6	4	50	25.91	193
**TborMTT1**	4	2	0	6	2	0	34	30.09	113
**TborMTT2**	5	2	2	9	1	2	49	30.06	163
**TelliMTT1**	6	3	0	7	1	2	45	29.22	154
**TelliMTT2**	6	3	0	6	0	3	42	28.97	145
**TmalaMTT1**	6	3	0	8	2	1	48	29.63	162
**TmalaMTT2**	5	3	0	8	1	2	44	27.16	162
**TmalaMTT3**	2	2	0	12	2	6	46	25.70	179
**TmalaMTT4**	1	1	0	7	1	3	25	25.25	99
**TpatMTT1**	4	2	0	6	2	0	34	29.06	117
**TpatMTT2**	4	2	0	6	3	3	39	26.35	148
**TamerMTT1**	4	2	0	5	1	1	31	29.25	106
**TamerMTT2**	6	3	0	8	2	1	48	30.57	157
Total Cys^(b)^	351	183	9	378	116	45	1082	28.27	147
% Cys ^(c)^	32.4	16.9	0.8	34.9	10.7	4.1	100		
**CuMT**									
TpigMT-2	0	0	0	1	12	2	28	29.17	
TtheMTT2	0	0	0	0	15	2	32	29.63	96
TtheMTT4	0	0	0	0	15	2	32	29.63	108
TrosMTT2	0	0	0	1	9	2	22	28.21	108
TtroMT1	0	0	0	1	12	2	28	28.00	78
TtroMT2	0	0	0	0	15	2	32	26.63	100
sp1.7-MT1	0	1	0	0	14	1	32	26.63	108
**TelliMTT6**	0	0	0	0	21	2	44	29.73	108
**TelliMTT8**	0	0	0	0	12	2	26	28.89	148
**TmalaMTT5**	0	0	0	0	15	2	32	29.63	90
**TborMTT3**	0	0	0	1	21	0	44	27.85	108
**TborMTT4**	0	0	0	1	6	2	16	26.67	158
**TborMTT6**	0	0	0	1	21	0	44	27.85	60
**TborMTT7**	0	0	0	0	21	0	42	28.19	158
**TborMTT8**	0	0	0	1	9	2	22	28.21	149
**TamerMTT3**	0	0	0	0	14	3	31	28.97	78
**ImMTT2**	0	0	0	0	27	0	54	32.34	107
Total Cys^(b)^	0	3	0	14	518	26	561	28.60	167
% Cys^(c)^	0	0.5	0	2.5	92.3	4.6	100		113

New MTs reported in this study are in bold text. A cluster is defined as any group of contiguous residues in which any two Cys residues are separated from one another by, at most, any other amino acid (X). ^(a)^ Percentage of Cys residues in the complete MT. ^(b)^ Total Cys residues/cluster type. ^(c)^ Percentage of Cys residues/cluster type

Another structural feature differentiating both *Tetrahymena* MT subfamilies is the relation between Lys and Cys residues along the polypeptide backbone. Lys residues also seem to have an important role in these proteins because Cys reactivity depends on the proximity of these basic amino acids. In ciliate CuMTs (subfamily 7b), as in mammalian MTs, Lys (K) residues are usually contiguous to Cys residues. Furthermore, the great majority of CXC motifs in CuMTs are CKC clusters (Fig. 2). On the other hand, in *Tetrahymena* CdMTs (subfamily 7a) the CKC clusters are less frequent and normally limited to the C-terminal regions of the type 2 submodules (Fig. 3). At physiological pH, Lys residues are positively charged, and it has been reported [31] that the presence of these adjacent basic residues considerably decreases the pK value thus decreasing the reactivity of Cys residues. Therefore, it is possible that *Tetrahymena* CuMTs may have a certain metal buffering capacity that differs from the one theoretically assigned to them.

### Modular/submodular structure of the subfamily 7a (CdMTs)

**Fig. 4:**
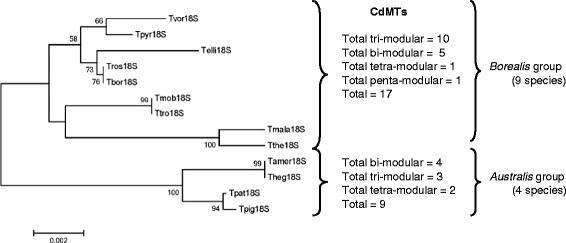
Phylogenetic tree (18S rRNA) of *Tetrahymena* species with reported CdMTs and their modular structures. Two well defined ribo-groups corresponding to both taxonomical groups were obtained. Multiple alignments of ribo-nucleotide sequences were obtained by using the T-Coffee program. Numbers indicate bootstrap values from 2000 replicates. Branch lengths are drawn to scale as indicated by the scale bar. The number of bi-, tri-, tetra- or penta- modular structures of CdMTs are indicated for each *Tetrahymena* group. See Table 1 for ciliate species identification

The highly conserved Cys residue locations in *Tetrahymena* CdMT sequences define a strict modular/submodular structure in all of these proteins [19] (Fig. 3). Each module was initially defined by the rule that every segment (with several exceptions) carries a CXCCX motif at its C-terminus, the last “X” being a Lys (K) or Glutamine (Q) residue (Fig. 3). Module lengths vary from 32 to 63 amino acids and are separated by linkers of 2–9 amino acids (Fig. 3). The number of modules per MT molecule is from 2 to 5, so at present 9 CdMTs have only two modules (bi-modular structure): TpyrMT-1, TtheMTT5, TrosMTT1, TpigMT-1, ThegMT1, TborMTT1, TmalaMTT4, TpatMTT1 and TamerMTT1. The majority (13) have a tri-modular structure: TtheMTT1, TtheMTT3, TtroMTT1, TvorMT1, TmobMT1, ThegMT3, TborMTT2, TelliMTT1, TelliMTT2, TmalaMTT1, TmalaMTT2, TpatMTT2 and TamerMTT2; only 3 of them (TmalaMTT3, ThegMT2 and ThegMT4) have four modules (tetra-modular), and only one (TpyrMT-2) has five modules (penta-modular) (Fig. 3). As shown in Fig. 4, the 18S rRNA phylogenetic tree of the 13 *Tetrahymena* species analysed separates them into *Borealis* and *Australis* groups. Although the species analyzed from the *Australis* group are still scarce, we detect a certain tendency towards a CdMT tri-modular structure in the *Borealis* group species with respect to the *Australis* group (Fig. 4). This could mean that the CdMT tri-modular structure might be more similar to the ancestral CdMT common structure for the *Tetrahymena* genus.

These modules are made up of two types of submodules: Type 1 submodules (sm1) have the consensus sequence C_2-3_X_6_C_1-2_X_6_ (with few exceptions), while complete Type 2 submodules (sm2) can be represented as C_2_X_6–8_ + CXCXXC_1-2_XXC_1-2_X_1–2_ (Fig. 3). Sm2 represents approximately the final half of the Type 1 submodules (C_2_X_6–8_), plus a quite conserved C-terminal region, where the last “X” is K (Lys) ~ 55 %, Q (Gln) ~ 36 %, E (Glu) ~ 7 % or N (Asn) ~ 1.5 %. Approximately 47 % of sm2 are incomplete; 42 % only present the C-terminal region, while ~ 5 % of them have only half the sm1 (½ sm1) (Fig. 3). In most cases (~54 %), these modules are made up of two complete sm1 and one complete sm2, but a ~ 34 % of them present incomplete modules, such as TpyrMT-2 (with four modules formed by only one sm1 and half of sm2). Despite the fact that *Tetrahymena* CuMTs do not present such clear modular structure, their consensus sequence CKCX_2-5_CXC is repeated multiple times (where in a few cases Lys (K) may be substituted by another amino acid) (Fig. 2). Therefore, a structural organization based on these repeats should also be considered [13].

Gene duplication is one of the main phases in the generation and evolution of new genes and seems to be the main mechanism involved in the evolution of these proteins, as several authors have noted [32–34]. Given the clear modular structure of *Tetrahymena* CdMTs and the highly conserved Cys repeats in both subfamilies, we might suppose that successive gene duplication events and subsequent specialization could be the main mechanisms involved in the evolution of these ciliate MTs. Therefore, the gene duplication hypothesis, already considered for *Tetrahymena* MTs [12, 13, 19, 26, 27], is the most credible mechanism for explaining the evolution of these proteins. A model that attempts to explain the evolutionary history of *Tetrahymena* MTs has been described in [12]. It is based on the hypothesis that both MT subfamilies were constructed from an ancestral module, containing the C_3_X_6_ motif (half of sm1), which was duplicated many times as a result of episodes of gradual environmental pollution. Therefore, both paralog duplications into the same species (several CdMT or CuMT gene isoforms are present in each species) and many orthologs could have been created in different related *Tetrahymena* species, giving rise to those MT isoforms that are currently known to us.

**Fig. 5:**
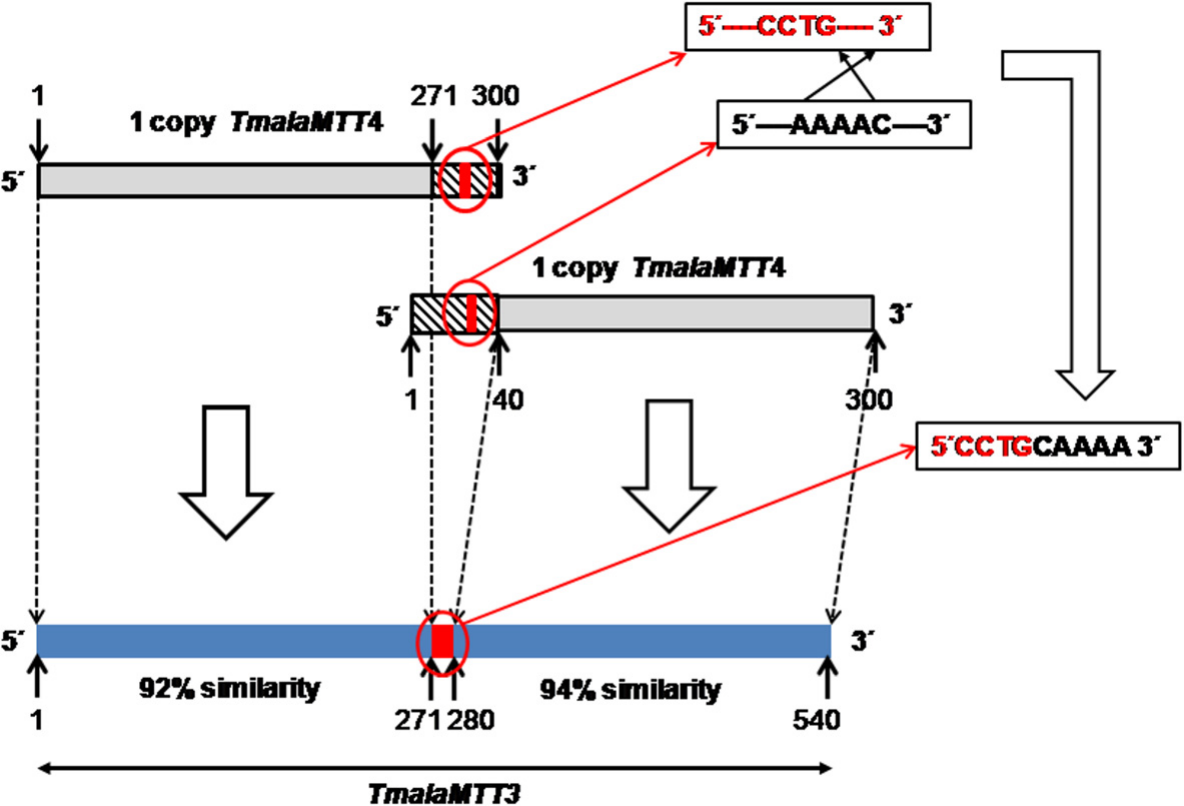
Schematic representation of *TmalaMTT3* gene origin from *TmalaMTT4* gene duplication. 5’ and 3’ striped sections show the eliminated regions (after recombination) of both *TmalaMTT4* gene copies. The red boxes in the striped sections from each *TmalaMTT4* gene copy and in the *TmalaMTT3* gene represent nucleotides forming the junction between both copies. Numbers indicate the nucleotide location. See the text for explanation

An example of a probable semi-complete duplication of a CdMT gene originating a new CdMT isoform in the same species, can be observed in CdMT genes from *T. malaccensis* (Fig. 5). Two copies of the *TmalaMTT4* gene isoform (300 bp of length) seem to have been involved in the creation of the *TmalaMTT3* gene (540 bp). A recombination process seems to have occurred between a 29 bp region from the 3’ end of one *TmalaMTT4* gene copy and a 40 bp region from the 5’end of a second copy of this same gene, together with the elimination of 60 bp from both gene copies, resulting in the origination of the junction area (9 bp) between both *TmalaMTT4* gene copies (Fig. 5). This 9 bp new sequence is formed by four nucleotides from one *TmalaMTT4* copy (3’end) and 5 inverted nucleotides from the other gene copy (5’end). Therefore, the *TmalaMTT3* gene is composed of a first uncompleted copy (271 bp) of the *TmalaMTT4* gene, a junction region (9 bp) originated after recombination and elimination, and a second uncompleted copy (260 bp) of the *TmalaMTT4* gene (Fig. 5). Through evolution, these uncompleted *TmalaMTT4* copies have suffered some degree of gene diversification, so each of them currently presents 92 and 94 % identity, respectively, with respect to the corresponding section of the original *TmalaMTT4* gene sequence (Fig. 5).

### Asymmetry of the codon usage for glutamine residues: phylogenetic implications

Glutamines (Q) are important residues for MTs because they are also involved in stabilizing the metal-protein complex [35]. Ciliates use a particular genetic code, because the UAA and UAG codons, which are universal stop codons in many organisms, codify the amino acid glutamine (Gln), UGA being the only stop codon used by these eukaryotic microorganisms (barring some exceptions) [36, 37]. Almost all ciliate MTs (except TpyrMT-1) contain Q residues (from 2 in TborMTT4 to 17 in ThegMT-4) (Table 3). In general, *Tetrahymena* CdMTs have more Q residues than CuMTs (Table 3). In a previous study [12] it was hypothesized that Gln residues were only codified by CAA codons in *Tetrahymena* CuMTs. However, after analyzing 10 new ciliate CuMTs, we have detected that this is not necessarily the case. Although the universal CAA codon is predominant in CuMTs (61.32 %), it is not the only one encoding Gln (29.24 % UAA, 7.55 % UAG and 1.89 % CAG) (Table 3). On the other hand, *Tetrahymena* CdMTs mainly use the UAA codon to codify this amino acid (53.93 %), together with other codons (29.21 % CAA; 17.17 % UAG and 1.69 % CAG) (Table 3). Moreover, in CdMTs these UAA codons are primarily located in liker regions (30.3 %) and at the end of sm2 (40.5 %). The total ratio of non-canonical (UAA + UAG)/canonical (CAA + CAG) Gln codons for *Tetrahymena* CdMTs is 123/55 = 2.23 (that is, almost twice as many non-canonical as canonical codons). The opposite is true for CuMTs, however, where this ratio is 39/67 = 0.58 or the inverse 67/39 = 1.71 (almost twice as many canonical as non-canonical codons). Interestingly, only two CdMTs sequences (TtheMTT3 and ThegMT4) use the four possible Gln codons (Table 3). This drastic asymmetry in codon usage for Gln residues again corroborates the fact that *Tetrahymena* CuMTs are more similar to standard MTs. Likewise, it shows another important difference between both MT subfamilies.

**Table 3:** Distribution of glutamine codons in both ciliate MT subfamilies

CdMT	Glutamine codons				
	CAA	UAA	UAG	CAG	Total
TpigMT-1	3	1	0	0	4
TpyrMT-1	0	0	0	0	0
TpyrMT-2	2	10	2	0	14
TtheMTT1	2	4	1	0	7
TtheMTT3	2	5	1	1	9
TtheMTT5	2	1	0	0	3
TrosMTT1	2	2	0	0	4
TtroMTT1	3	1	0	0	4
TmobMT1	4	1	0	0	5
TvorMT1	4	0	0	0	4
ThegMT1	2	2	0	0	4
ThegMT2	2	4	2	0	8
ThegMT3	1	3	0	1	5
ThegMT4	1	10	5	1	17
**TborMTT1**	2	4	0	0	6
**TborMTT2**	3	3	1	0	7
**TelliMTT1**	1	6	2	0	9
**TelliMTT2**	3	5	3	0	11
**TmalaMTT1**	1	5	1	0	7
**TmalaMTT2**	3	3	1	0	7
**TmalaMTT3**	2	5	0	0	7
**TmalaMTT4**	0	5	0	0	5
**TpatMTT1**	2	4	1	0	7
**TpatMTT2**	2	4	5	0	11
**TamerMTT1**	1	4	1	0	6
**TamerMTT2**	2	4	1	0	7
**Total (%)** ^**a**^	52 (29.21 %)	96 (53.93 %)	27 (15.17 %)	3 (1.69 %)	178 (100 %)
**CuMT**					
TpigMT-2	6	0	0	0	6
TtheMTT2	4	0	0	0	4
TtheMTT4	4	0	0	0	4
TrosMTT2	5	0	0	0	5
TtroMT1	5	0	0	1	6
TtroMT2	2	0	1	1	4
Sp1.7-MT1	3	2	2	0	7
**TelliMTT6**	5	1	0	0	6
**TelliMTT8**	3	2	0	0	5
**TmalaMTT5**	3	1	0	0	4
**TborMTT3**	4	7	2	0	13
**TborMTT4**	2	0	0	0	2
**TborMTT6**	4	7	2	0	13
**TborMTT7**	5	2	1	0	8
**TborMTT8**	5	0	0	0	5
**TamerMTT3**	4	0	0	0	4
**ImMTT2**	1	9	0	0	10
**Total (%)** ^**a**^	**65 (61.32 %)**	**31 (29.24 %)**	**8 (7.55 %)**	**2 (1.89 %)**	**106 (100 %)**

New MTs reported in this study are in bold text.^a^ Total glutamine residues using a type of codon and percentage

From a phylogenetic point of view, which we reported in a previous review article [12], it seems CuMTs are diverged earlier than CdMTs as it has been reported that rather than resulting from a single ancient event, these genetic code deviations arose independently several times within the phylum Ciliophora [36, 37]. Also, if these changes occurred in the tRNA(Gln) gene(s) of an ancestral ciliate using the standard genetic code, we would have to assume that *Tetrahymena* CuMTs appeared before CdMTs (which use mainly UAA codons). This agrees with the assumption, supported by several authors, that two early MT lineages (Cu and Zn thioneins) were already present in the early phases of eukaryotic evolution [32]. It appears that early on in evolution, the primary function of MTs was to bind to physiologically important or essential metals (such as Cu or Zn). Accordingly, the appearance and evolution of MTs was probably not dictated by Cd, although with the evolution of higher life forms, MTs became more indispensable for protecting against Cd and other non-essential metals than for performing other suggested functions [4].

### Other interesting features of *Tetrahymena* MTs

*Tetrahymena* MTs are considerably longer (60–193 aa) than standard ones (25–82 aa). Of the ciliate CdMTs and CuMTs, the longest are ThegMT4 (193 aa) and ImMTT2 (167 aa) or TborMTT3 and TborMTT6 (158 aa each), respectively (Table 2). The length they reach is therefore more than double that of the longest standard MTs. Despite having the longest MTs and a higher number of Cys residues, the Cys % lies within the range for standard MTs (16–32 %) (Table 2). However, because of their greater amount of Cys residues, they present a higher theoretical metal binding capacity than standard MTs. The calculation of this theoretical metal binding capacity takes into consideration that all Cys residues in vertebrate MTs are involved in heavy metal binding; therefore, the stoichiometry is Cd_7_(Cys)_20_ for CdMTs and Cu_12_(Cys)_20_ for CuMTs [4]. The 3D structure analysis of several MTs corroborates this stoichiometry [38]. We can assume that this stoichiometry is also applicable to *Tetrahymena* MTs and have calculated the theoretical binding capacity for all of them (Additional file 1). The shortest *Tetrahymena* CdMTs (TtheMTT5 and TmalaMTT4) (Table 2) are able to bind 8 or 9 Cd^2+^/molecule and up to 19 Cd^2+^/molecule (Additional file 1) in the case of TpyrMT-2, which is one of the longest CdMT (181 aa) with the highest amount of Cys residues (54 residues) (Table 2). The average amount of the theoretical Cd binding capacity of these CdMTs is about 15 Cd^2+^/molecule (Additional file 1), which is more than double that of standard MTs (7 Cd^2+^/molecule). Some of these data have been corroborated experimentally; for instance: 12 Cd^2+^ per mole of protein or 11 Cd^2+^ per polypeptide for TpyrMT-1 [39]. Likewise, a stable in vitro Cd_16_(Cys)_48_ complex has been suggested [40] for TtheMTT1 (a similar value to the theoretical one assigned to this CdMT) (Additional file 1), and Cd_11_(Cys)_32_ complex has also been suggested [40] for the CuMT TtheMTT2, with a metal-to-Cys residue ratio of about 1:3 for both *T. thermophila* MTs. In addition, it was found that Cu^2+^ cannot replace Cd^2+^ from the Cd_16_-TtheMTT1 complex, but Cu^2+^ can replace Cd^2+^ from the Cd_11_-TtheMTT2 complex [40]. This confirms the classification of the TtheMTT1 and TtheMTT2 as CdMT and CuMT, respectively. More recently, we have analysed the metal binding preference and abilities of the five MT isoforms from *T. thermophila* by electrospray mass spectrometry, circular dichroism and UV–vis spectrophotometry [25]. We conclude that both CdMT isoforms (TtheMTT1 and TtheMTT5) which yield unique Cd_17_- and Cd_8_- complexes, respectively, are optimal for Cd^2+^ coordination, thus corroborating the theoretical metal binding capacity values obtained for these CdMTs (Additional file 1). The MTT3 isoform showed poor binding abilities with both Cd^2+^ and Cu^+^, and yielded the best result whith Zn^2+^. Although this MT, also considered as a CdMT, has a theoretical metal binding capacity value of about 15, the two Histidine (His) residues present in the protein enhance the relative affinity for Zn^2+^ through their imidazol rings [41, 42] in comparison with Cd^2+^. His residues, the most frequent Zn ligands in metaloenzymes, are also present in MTs (1–4 residues/molecule) from a variety of species (bacteria, fungi, plants and animals), thus increasing the affinity for this essential metal. Of the *Tetrahymena* MTs, only two CdMTs (TtheMTT3 and TpatMTT2) (Fig. 3) contain His residues, while CuMTs contain nine isoforms with one or two His (Fig. 2), coinciding with the general knowledge that this amino acid is more abundant in CuMTs than in CdMTs. In addition, His residues have an important role in MT proteins as they stabilize the formation of metal-protein complexes [43]. Both *T. thermophila* CuMTs (TtheMTT2 and TtheMTT4 isoforms), were found to form homometallic Cu-complexes (mainly Cu_20_-MT) [25], which coincides with the theoretical metal binding capacity value obtained (19 Cu ions/molecule) (Additional file 1). Zn-MT complexes were only found in TtheMTT4 (mainly Zn_10_-MTT4) [25]. These CuMTs (TtheMTT2 and TtheMTT4) differ in only one relevant amino acid position (Asn^89^/Lys^89^), and it is interesting how this amino acid position change increases the CuMT character (higher affinity for Cu ions) of TtheMTT2 (Asn) in relation to TtheMTT4 (Lys) [25]. This special feature has also been reported in snail MTs, balancing in favour of Zn/CdMTs (Lys) or CuMTs (Asn) [44].

Another peculiar characteristic of MTs (including those from *Tetrahymena*) is the large asymmetry in the ratio of specific amino acids, such as the positively charged amino acids Lys and Arg. There is a considerably higher use of Lys residues with respect to Arg residues, and this asymmetry (Lys > > Arg) appears in all reported MTs. In ciliate MTs the Arg residues are almost absent of the CdMTs, only two (TtheMTT3 and ThegMT2) present one Arg residue (Fig. 3), and only two CuMTs (TborMTT3 and ImMTT2) have Arg residues, with one and two residues, respectively (Fig. 2). The location of these Arg residues is the same as those of Lys residues in other ciliate MT sequences. These two positively charged amino acids (Lys and Arg) are mostly exposed to the protein surface and play important roles in protein stability by forming electrostatic interactions. Arginine forms a higher number of electrostatic interactions compared to lysine. Experiments carried out on the green fluorescent protein (GFP), after changing lysine for arginine on the protein surface and retaining protein activity, have shown that the GFP variant was relatively more stable compared to the control GFP (in the presence of urea, basic pH or ionic detergents), but the thermal stability of the protein was similar to the control [45]. On the other hand, proteins with elevated solubility, a higher expression and abundant intracellular levels have an increased ratio of Lys content to Arg content [46]. In general, MTs have large number of Lys residues and null or very few Arg residues per molecule, preferring higher solubility and the avoidance of protein aggregates to higher stability by forming a larger number of electrostatic interactions (salt-bridges or hydrogen bonds). This could therefore mean that the asymmetry (Lys > > Arg) reported in all MTs is a basic requirement for forming proteins with an elevated solubility and high intracellular level to respond to metal stress.

### Analysis of *Tetrahymena* MT cDNAs

After comparing all new isolated cDNAs of putative MT genes with the genomic DNA from the corresponding *Tetrahymena* species, we confirmed that none of these genes have introns in their open reading frames, which is also the case in all previously reported *Tetrahymena* MT genes [13]. The absence of introns could be related to a faster gene response to different environmental stressors. The presence of introns can delay regulatory responses and they are selected against in genes with transcripts requiring rapid adjustment in order to survive environmental changes [47]. In general, the gene expression of *Tetrahymena* MT genes is very fast, as reported in a CdMT gene (*MT-1*) from *T. pigmentosa*, in which an approximately tenfold increase of this transcript was detected after 30 min Cd treatment [15]. An exception was reported in the gene isoform *MTT5* of *T. thermophila*, in which an intron was located in the 3’UTR of the corresponding cDNA. The existence of *MTT5* mRNAs with and without this intron supports the first case of alternative intron splicing reported in this ciliate [19]. In the *Tetrahymena* Comparative Database (Broad Institute, Cambridge, USA) the *MTT3* DNA sequence from *T. malaccensis* (registered as EIA_07390.2 hypothetical protein) shows a putative intron (60 nucleotides). After a more detailed analysis, we detected that the putative intron corresponds to the amino acid sequence of the second type 1 submodule from the second module, sequence that is quite conserved in almost all *Tetrahymena* CdMTs. The nucleotide sequence identified as an intron (EIA_07390.2) starts in GT and ends in AG (with a 68.3 % A + T), and therefore, it might be assumed that it would coincide with the ciliate consensus intron ends (5’GTAAG/TAG 3’). A similar situation in which no introns are observed is detected in *TamerMTT1* [KU052681] and *TpatMTT1* [KU167652] cDNAs. The GT pair corresponds to a Cys residue (tgt codon), while AG pair corresponds to Glu (E) residue (gag codon). The same occurs in the TamerMTT1 and TpatMTT1 CdMT sequences, but not in the rest of the *Tetrahymena* CdMTs, because the majority codon used for Glu residues is gaa. In addition, the A + T content of these regions has very similar values to the complete ORF sequences from all CdMTs reported to date (an average of 60.5 %). All of this corroborates that the putative intron reported in a CdMT from *T. malaccensis* (EIA_07390.2) is not a real intron but rather an error.

In general, MT genes are mainly regulated at transcriptional level [48]. As in a previous study using an *in silico* analysis [19], we identified several conserved motifs in both 5’ and 3’UTR regions from the new isolated cDNA molecules, which may be related to the regulation of their gene expression and/or transcript processing. In the 5’UTR region or putative promoter we identified two types of motifs (Tables 4 and 5): a TATA box (TAATAA) with an average number of ≈ 3 motifs/cDNA in both Cd- and CuMTs, and motifs similar to MTCM1 [19] with an average number of ≈ 5 motifs/cDNA in CdMTs or ≈ 3 in CuMTs. This MTCM1 motif was identified in almost all *Tetrahymena* MT promoters (Tables 4 and 5), so these sequences might play an important role in the gene expression regulation of these MTs. Most MTCM1 motifs include the consensus sequence TGA(N)TCA or similar (where “N” means any nucleotide), which recalls to the sequence (TGA(G/C)TCA) binding the eukaryotic AP-1 transcription factors [49]. In *Saccharomyces cerevisiae* an AP-1 transcription factor (YAP-1) is involved in response to oxidative stress and metal resistance [50]. Transcription factors similar to AP-1 have been detected in the MT promoters of insects (*Drosophila melanogaster*) and mollusks (*Crassostrea virginica*) [51]. AP-1, also known as c-jun, is a member of the bZIP superfamily of eukaryotic DNA-binding transcription factors. In *T. thermophila* the number of MTCM1 motifs in the three CdMT putative promoters is correlated with the gene expression level of each gene therefore, the relative induction ranking of gene expression is: *TtheMTT5*> > *TtheMTT1* > *TtheMTT3* [19]. This motif is present 6 times in *TtheMTT1*, twice in *TtheMTT3* and 13 times in *TtheMTT5* promoter region (Table 4). The promoter of the *TtheMTT5* gene has a 416 bp tandem duplication (with 96 % identity to each other). Five copies of the MTCM1 motif are present in each duplication and another three copies are near to the start codon [19]. Quantitative gene expression analysis revealed that *TtheMTT5* is the gene most strongly induced by diverse environmental stressors (*TtheMTT5 > > TtheMTT1 > TtheMTT3*) [19]. The presence of the duplicated promoter region in *TtheMTT5* may be related to the high expression level of this CdMT gene when compared with the rest of *T. thermophila* CdMT genes. There is no direct and clear correlation between the number of MTCM1 motifs in the putative promoter regions of each *Tetrahymena* MT gene studied and their higher or lower expression level.

**Table 4:**
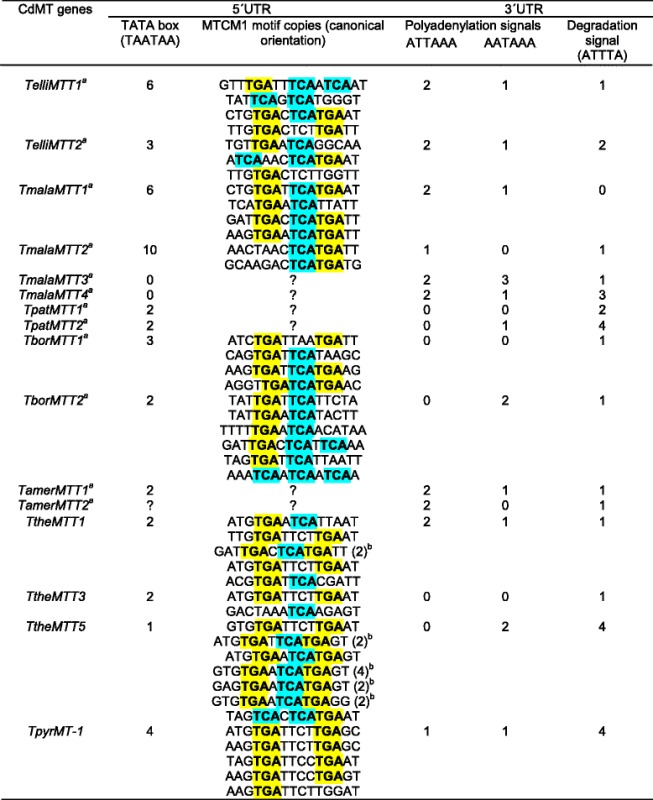
Conserved motifs detected in 3’ and 5’ UTR regions of *Tetrahymena* CdMT genes

^a^New CdMT genes reported in this study. ^b^ Numbers in parenthesis indicate the copy number/motif. TCA and TGA trinucleotides are highlighted in blue and yellow to facilitate motif sequence comparisons and identifications of the AP-1 binding related element (TGANTCA). ? : unknown

**Table 5:**
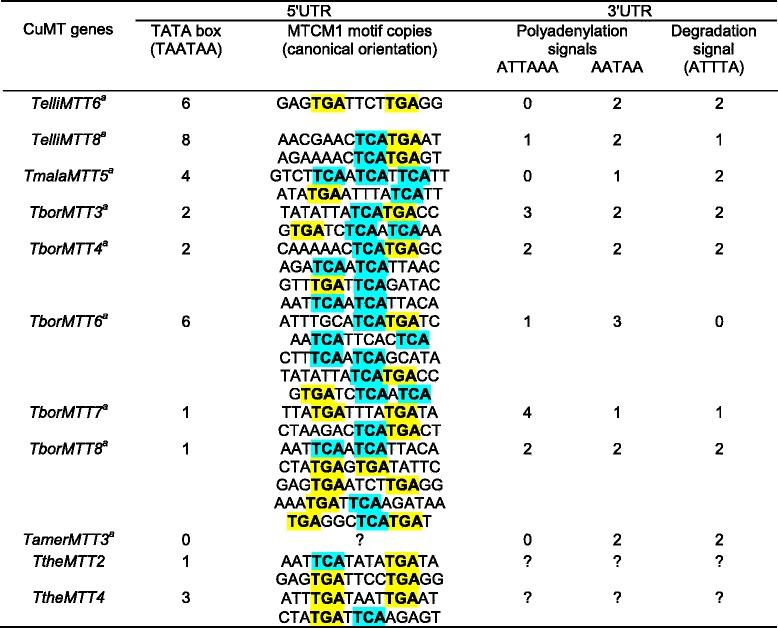
Conserved motifs described in 3’ and 5’UTR regions of *Tetrahymena* CuMT genes

^a^New CuMT genes described in this paper. TCA and TGA trinucleotides are highlighted in blue and yellow to facilitate motif sequence comparisons and identifications of the AP-1 binding related element (TGANTCA). ? : unknown

Two types of polyadenylation signals were detected on 3’UTR regions of these cDNAs (Tables 4 and 5). The average number of these polyA sites is about 3.2 for CdMTs, and 3.9 for CuMTs. Likewise, mRNA degradation signals (ATTTA) appear in almost all MT genes analyzed, with an average number of 1.7 (Tables 4 and 5).

### Comparative analysis of the MT gene expression under several environmental stressors

Of the 21 new *Tetrahymena* MT genes only 6 were analyzed, using qRT-pCR, owing to the great similarity among MT gene nucleotide sequences isolated from the same *Tetrahymena* species, which makes it impossible to design specific primers to differentiate each gene. We studied the expression of two CdMT genes (*TborMTT1* and *TborMTT2*) and one CuMT gene (*TborMTT7*) from *T. borealis*. The rest of the MT genes isolated from this species have a great similarity; the *TborMTT3*/*TborMTT6* and *TborMTT4/TborMTT8* pairs have 96 and 98 % identity, respectively. One CuMT gene (*TelliMTT6*) from *T. elliotti* was analyzed because the *TelliMTT1/TelliMTT2* pair has 85 % identity, and although *TelliMTT8* is very similar to *TelliMTT6* the later is much longer, making it possible to design specific primers to amplify a fragment of the *TelliMTT6* 3’ region. Only one CuMT gene(*TamerMTT3*) was analyzed from *T. americanis* because *TamerMTT1* and *TamerMTT3* are almost identical (99 %). Finally, one other CuMT gene expression (*TmalaMTT5*) was carried out from *T. malaccensis* because the *TmalaMTT1/TmalaMTT2* and *TmalaMTT3/TmalaMTT4* pairs have 84 and 94 % identity, respectively. Chang et al. (2014), used qRT-PCR to analyze the expression of a gene (*TmalMT1*) isolated from *T. malaccensis* under metal stress (Cd^2+^, Zn^2+^, Cu^2+^ or Pb^2+^) [16], but this gene (which is the same as our *TmalaMTT1* gene) has 84 % identity with a second CdMT gene, *TmalaMTT2*, which we isolated from the same *Tetrahymena* species, this being the reason we were not able to analyze them by qRT-PCR. Therefore, the gene expression analysis carried out by Chang et al. (2014) [16] very probably corresponds to the sum of both CdMT gene expressions (*TmalMT1* = *TmalaMTT1* + *TmalaMTT2*). In the case of *T. patula* it was not possible to analyze the expression of any of the genes because both CdMT genes (*TpatMTT1* and *TpatMTT2*) have 91 % identity.

**Fig. 6:**
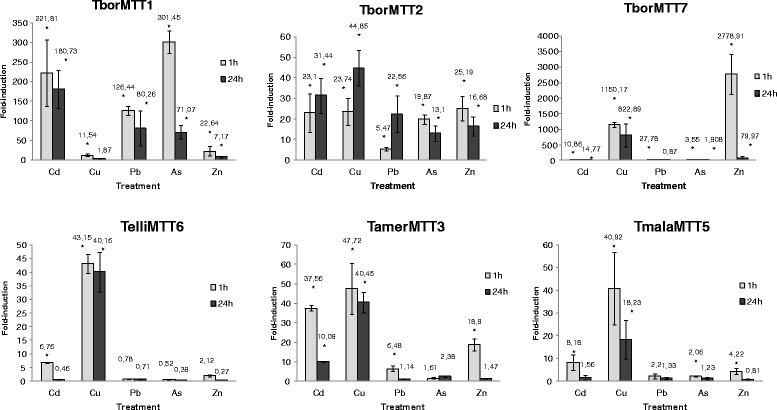
qRT-PCR analysis of six *Tetrahymena* MT genes after diverse metal treatments. Relative expression levels for each MT gene are shown in the different histograms, after cellular treatment with heavy metals, during exposures of 1 h (grey bars) or 24 h (black bars). Each bar of the histogram corresponds to an average value ± SD of two or three independent experiments. Asterisks indicate significant differences from control at *p* < 0.05. Numbers on each bar indicate the corresponding average fold induction value for each treatment. See Table 1 for MT gene identification

Figure 6 shows the relative fold induction values for each MT gene expression under five different metal(loid) stress treatments at 1 h (short exposure) or 24 h (long exposure). In general, the induction values are higher at 1 h of treatment than after 24 h of metal(loid) exposures, except for the CdMT gene *TborMTT2* under Cd^2+^, Cu^2+^ or Pb^2+^ treatments (Fig. 6). Both putative CdMT genes from *T. borealis* (*TborMTT1* and *TborMTT2*) are induced by Cd^2+^ among other inorganic cations, but there are several differences in the expression patterns of each gene isoform. At both 1 and 24 h exposures of Cd^2+^, Pb^2+^ or As^5+^, the expression levels for *TborMTT1* are considerably higher than for the *TborMTT2* gene. On the other hand, the *TborMTT2* gene isoform seems to be induced later and more uniformly with these metal(loid)s. Therefore, under the same stress conditions, two differential gene expression behaviors are detected for these *T. borealis* CdMT isoforms; the *TborMTT1* gene responds faster and with a higher level to most metal(loid) stress, while the *TborMTT2* gene responds later and with a lower level under the same stress conditions (Fig. 6). Both genes respond to arsenate (As^5+^). After 1 h of treatment with this toxic metalloid, *TborMTT1* is induced about 300-fold while *TborMTT2* is only induced ≈ 20-fold. The toxicity of arsenic depends on its chemical form and state of oxidation, and is generally assigned to its capacity to produce ROS (reactive oxygen species). Arsenic-induced ROS originate lipid peroxidation and genotoxic damage [52]. Metallothioneins reduce arsenic toxicity, either because As^5+^ may interact with their thiol groups and to involve detoxification, or they may act as antioxidants to protect against the oxidative stress originated by As^5+^ [53]. In fact, arsenic induces MT gene expression [54], including some previously studied *Tetrahymena* MT genes (Table 6) [19, 23].

**Table 6:** Ranking of relative fold-induction values of *Tetrahymena* MT genes obtained by qRT-PCR analysis after different heavy metal treatments

MT gene	Subfamily	Relative fold-induction value ranking	Treatment	Reference
*TpyrMT-1*	CdMT	Cd > Cu > Zn > Hg	1 h	[12]
*TpyrMT2*	CdMT	Cd > Cu > Zn > Hg	1 h	[12]
*TtheMTT1*	CdMT	Cd > Hg > Cu > Zn	30 min	[14]
		Cd > Zn > Pb > Cu > Ni	1 h	[2]
		Cd > Pb > As > Cu > Zn > Ni	24 h	
*TtheMTT3*	CdMT	Zn > Cd > Pb > Ni > Cu	1 h	[2]
		Cd > Zn > As > Ni > Cu > Pb	24 h	
*TtheMTT5*	CdMT	Pb > Cd > Zn > Cu > Ni	1 h	[2]
		Pb > As > Cd > Cu > Zn > Ni	24 h	
*TrosMTT1*	CdMT	Cd > Pb > As > Cu > Zn	1 h	[17]
		Pb > Cd > As > Zn > Cu > Ni	24 h	
*TpigMT-1*	CdMT	Hg > Pb > Cd > Cu > Zn	1 h	[41]
*ThegMT1*	CdMT	Zn > Pb > Cd > Cu	1 h	[19]
		Pb > Cd > Zn > Cu	24 h	
*ThegMT2*	CdMT	Zn > Cd > Cu > Pb	1 h	[19]
		Pb > Zn > Cd > Cu	24 h	
*TmalMT1*	CdMT	Cd > Zn > Cu > Pb	1 h	[19]
		Cd > Zn > Cu > Pb	24 h	
*TmobMT1*	CdMT	Cd > Cu > Pb > Zn	1 h	[19]
		Cd > Pb > Cu > Zn	24 h	
*TborMTT1*	CdMT	As > Cd > Pb > Zn > Cu	1 h	This paper
		Cd > Pb > As > Zn > Cu	24 h	
*TborMTT2*	CdMT	Zn > Cd ≈ Cu > As > Pb	1 h	This paper
		Cu > Cd > Pb > Zn > As	24 h	
*TrosMTT2*	CuMT	Cu > Pb > Cd > Zn > As	1 h	[17]
		Cu > Zn > Cd > Pb > Ni	24 h	
*TborMTT7*	CuMT	Zn > Cu> > Pb > Cd > As	1 h	This paper
		Cu > Zn> > Cd > As > Pb	24 h	
*TelliMTT6*	CuMT	Cu > Cd > Zn > Pb ≈ As	1 h	This paper
		Cu> > Pb > Cd ≈ As ≈ Zn	24 h	
*TamerMTT3*	CuMT	Cu > Cd > Zn > Pb > As	1 h	This paper
		Cu > Cd > As > Zn ≈ Pb	24 h	
*TmalaMTT5*	CuMT	Cu > Cd > Zn > Pb ≈ As	1 h	This paper
		Cu > Cd > Pb ≈ As > Zn	24 h	

The rest of *Tetrahymena* MT genes analyzed are putative CuMTs, which are induced primarity but not exclusively by Cu^2+^ (Fig. 6), as is also the case in other previously studied CuMT genes from other *Tetrahymena* species [23] (Table 6). The *TborMTT7* gene is enormously induced by Zn^2+^ and Cu^2+^, especially after 1 h of treatment. This is considerably more than other CuMT genes (Fig. 6), so this MT may play an important role in essential metal (Zn^2+^ or Cu^2+^) homeostasis, which might also occur in CdMTs, such as; TtheMTT3 [19], ThegMT1, ThegMT2 [16] and other CuMTs (Table 6). Almost all of these have His residues in their molecules (Figs. 2 and 3), which, as previously indicated, enhance the relative affinity for Zn^2+^ [41, 42] or Cu^2+^.

Comparisons of the qRT-PCR values obtained by different authors is difficult because of the different experimental conditions used, but even when conditions are the same and identical samples are used, it is difficult to reproduce experiments from different laboratories [55]. However, after the qRT-PCR values from different *Tetrahymena* MT genes are compared (Table 6), some general considerations can be inferred: a) In general, *Tetrahymena* CdMT genes are mainly induced by Cd^2+^ (Cd > Cu), whereas CuMT genes are induced by Cu^2+^ (Cu > Cd). This difference corroborates their separation in two previously well-defined sub-families (7a or CdMTs and 7b or CuMTs) [19]. b) Induction by Zn^2+^ sometimes occupies first position in the ranking of relative fold-induction values for both Cd- or CuMT genes but only with 1 h metal treatments, indicating a possible metal homeostatic role. c) Occasionally, induction by Pb^2+^ replaces Cd^2+^ at the top of the ranking, as in the case of *TtheMTT5, TrosMTT1, ThegMT1* and *ThegMT2*. This is likely to be due to similarities between Pb^2+^ and Cd^2+^ in their chemical structure. MTs are induced by Pb^2+^ in rats, humans and fishes [56–58]. Pb^2+^ is second to Cd^2+^ in its ability to displace Zn^2+^ from hepatic ZnMT and is able to displace Cd^2+^ from the CdMT complex [59, 60]. A transcriptome study in plants has revealed that many genes respond similarly to Pb^2+^ and Cd^2+^ [61]. Other metals can also induce *Tetrahymena* MT gene expression, for instance: La^3+^ induces the expression of both *TtheMTT1* and *TtheMTT2* genes. Fluorescence analysis shows that La^3+^ binds to both *T. thermophila* MTs [40], and that the *TpigMT-1* gene is induced by Hg^2+^ [26].

**Fig. 7:**
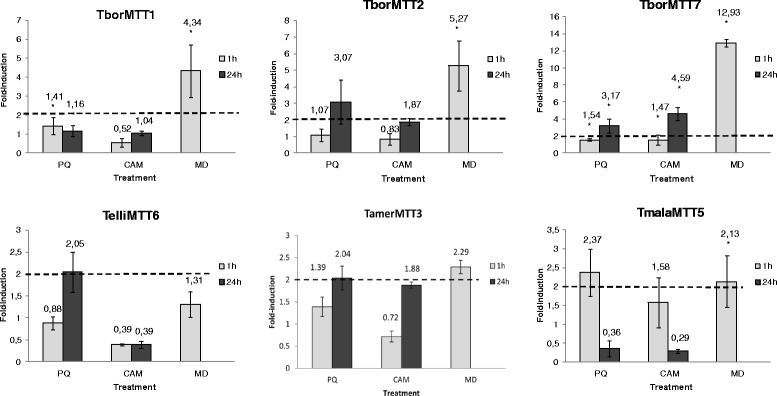
qRT-PCR analysis of six *Tetrahymena* MT genes after apoptotic or oxidative stress treatments. Relative expression levels for each MT gene are shown in the different histograms, after cellular treatment with different oxidative or apoptotic stressors, during exposures of 1 h (grey bars) or 24 h (black bars). Each bar of the histogram corresponds to an average value ± SD of two or three independent experiments. Asterisks indicate significant differences from control at *p* < 0.05. Numbers on each bar indicate the corresponding average fold induction value for each treatment. A gene expression induction is considered positive when the fold-induction value obtained is > 2 (indicated by the dashed line). See Table 1 for MT gene identification

MT gene expression, including both *Tetrahymena* Cd- and CuMT genes, is induced by oxidative stress originated by H_2_O_2_ or organic compounds, such as Paraquat (PQ) or Menadione (MD) [19–21, 23, 26]. However, the fold-induction values obtained after PQ treatment are generally very low or null (Fig. 7). On the other hand, fold-induction values for MD (1 h treatment) are generally significantly higher than those obtained with PQ treatment (Fig. 7). Both compounds are pro-oxidants generating superoxide anions through redox cycling [62, 63], and they are known to potentially activate the transcription of some MTs [64], such as CUP1 from *S. cerevisiae* [65]. Certain antioxidant ability for both *T. thermophila* MTs (TtheMTT1 and TtheMTT2) has been suggested, because of the appearance of disulfide bonds in CdMT complexes after in vitro reaction with NO [40]. However, as shown by both previous results and our own (Fig. 7), other *Tetrahymena* CdMT genes (*TpyrMT-2* and *TrosMTT1*) are not significantly induced by H_2_O_2_ or PQ [20, 23]. Likewise, the apoptosis inductor camptothecin (CAM) does not seem to be a good inducer of *Tetrahymena* MT gene expression, except in the case of the *TborMTT7* gene, which is slightly induced (≈4.5- fold) after 24 h treatment (Fig. 7).

**Fig. 8:**
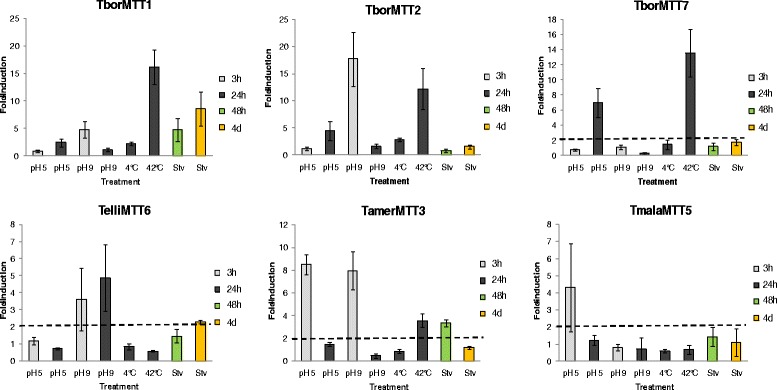
qRT-PCR analysis of six *Tetrahymena* MT genes after different abiotic stress treatments. Relative expression levels for each MT gene are shown in the different histograms, after cellular treatment with different abiotic stressors (pH, temperature or starvation (stv) during exposures of 3 h (grey bars), 24 h (black bars), 48 h (green bars) or 4 days (yellow bars). Each bar of the histogram corresponds to an average value ± SD of two or three independent experiments. Asterisks indicate significant differences from control at *p* < 0.05. Numbers on each bar indicate the corresponding average fold induction value for each treatment. A gene expression induction is considered positive when the fold-induction value obtained is > 2 (indicated by the dashed line). See Table 1 for MT gene identification

Other abiotic environmental stressors (pH, temperature and starvation) have been analyzed as inducers of *Tetrahymena* MT gene expression. As shown in Fig. 8, acid pH induces gene expression in some *Tetrahymena* MT species (at both 3 or 24 h treatments), mainly in CuMTs. Under basic pH stress, only four new *Tetrahymena* genes are induced (at 3 or 24 h treatments) with fold-induction values between ≈ 4.7 and 17.7. In general, pH changes induce a very wide and variable range of responses in *Tetrahymena* MT genes, from null [23] to a certain degree of induction irrespective of whether they are Cd- or CuMTs.

High temperature (42 °C) induces gene expression in the majority of *Tetrahymena* MT isoforms (Fig. 8). *T. rostrata* MTs seem to be quite sensitive to thermal stress, because both isoforms (Cd- and CuMT) are considerably induced by the 2 h heat-shock (42 °C) treatment [23]. Likewise, the three isoform genes analyzed from *T. borealis* are also induced by 42 °C (24 h treatment) (Fig. 8). According to several phylogenetic studies, both *Tetrahymena* species are very closely related [66] (Fig. 4) and both species belong to the RSA2 riboset of the *Borealis* group, although *T. rostrata* is a histophagous ciliate, whereas and *T. borealis* not [66].

However, other MT genes from the *Tetrahymena* species are not induced by high temperature [19] (Fig. 8), although it has been reported that the *TtheMTT1* gene is also induced (by about 8-fold) at 40 °C (30 min exposure) [21]. In general, a low temperature (4 °C) does not induce the gene expression of *Tetrahymena* MT genes.

One of the main and more common environmental stressor to which cells are subject is starvation. Among newly analyzed *Tetrahymena* MT genes, the *TborMTT1* gene has a higher fold-induction value, while the rest of the genes present very low or null induction values under starvation stress (Fig. 8). Previous studied of *Tetrahymena* MT genes have also shown induction (mainly after 24 h treatment) under starvation conditions [19, 23]. The *TtheMTT5* gene is expressed during ciliate conjugation (a sexual process induced and developed under starvation) [19], and the *S. cerevisiae CUP1* metallothionein gene is induced under glucose starvation [67].

At present, this multi-stress character has been reported by the majority of expression analyses of MT genes under diverse abiotic stressors [68, 69]. The transcriptional induction of several MT genes by a variety of stress conditions suggests that cellular exposure to one type of stressor might lead to the acquisition of tolerance towards another different stressor. This cross-protection reported in some organisms suggests at least the partial existence of overlapping between genome responses to different types of stress [70]. This overlapping genome expression comprises general stress-responsive genes, such as heat-shock or MT genes.

Independent of this general multi-stress feature of MTs, a differential gene expression level exists under the same environmental stressor, in different MT gene isoforms from the same subfamily, the same *Tetrahymena* species or among different species, which also seems to exist in different mammalian MT isoforms [1, 48]. There are several examples in the same *Tetrahymena* species (Table 6): a) the three CdMT isoform genes from *T. thermophila* present differential expression patterns. Both TtheMTT1 and TtheMTT5 seem to be general stress and metal detoxification proteins. However, the gene expression induction values reached by *TtheMTT5* gene are considerably higher than the rest of the *T. thermophila* MT isoforms [19], and it responds preferably but not exclusively to Pb^2+^. On the other hand, the *TtheMTT1* gene preferably responds, at a lower level, to Cd^2+^ [19]. In fact, according to a physicochemical analysis of these MTs recombinantly synthesized as metal-complexes [25], TtheMTT1 has a higher affinity or binding preference by Cd^2+^ than TtheMTT5 isoform. The transformed *T. thermophila* strain (GFPMTT5) with the *P*_*MTT1*_*::GFP::MTT5* plasmid construct (which includes the *TtheMTT1* promoter, the green fluorescent protein as a reporter gene, and the *TtheMTT5* open reading frame) has been shown to be about 10 times more resistant to Cd^2+^ with regard to the wild-type strain [71], indicating that the *TtheMTT1* promoter responds to Cd^2+^ and that an increase in the *TtheMTT5* gene dose (into a cytoplasm multi-copy plasmid) affects the Cd^2+^ LC_50_ value of this ciliate. A third His-containing CdMT isoform (TtheMTT3) has a higher affinity to Zn^2+^ [19], and could be involved in the intracellular homeostasis of this metal; b) two CdMT isoform genes from *T. hegewischi* (*ThegMT1* and *ThegMT2*) [16] show different gene expression induction patterns, although, as in other cases, these fold-induction values change depending on the metal time exposure (Table 6); c) likewise, the two new CdMT gene isoforms from *T. borealis* (*TborMTT1* and *TborMTT2*) also have different gene expression induction patterns (Table 6).

In the majority of organisms, several isoforms of MT genes are present, suggesting the existence of differential cellular roles for them. The differential gene expression patterns reported from different MT isoforms under the same environmental stressor corroborate this idea. However, more extensive analysis needs to be done on the molecular behavior of each MT isoform and its relevance with respect to cellular response to a specific stress. Future analyses of knockout strains in one MT gene isoform and expression studies carried out on the rest will be of great importance for understanding the role of each MT isoform.

## Conclusions

At present, a total of 42 MT gene isoforms from different *Tetrahymena* species have been reported, of which 21 have been isolated as new cDNAs and reported in this study. In addition, one more MT gene from the sequenced macronuclear genome of the *Tetrahymena*-related ciliate *Ichthyophthirius multifiliis* has been added to the ciliate MT analysis described in this paper. From analysis of the results, the following points can be conclude:

1. Two main *Tetrahymena* MT subfamilies (7a or CdMTs and 7b or CuMTs) were once more corroborated after the 21 new MT cDNAs isolated from different *Tetrahymena* species were analyzed. Both MT subfamilies are based on their protein structural organization (Cys residue patterns) and their preferential gene induction under Cd^2+^ or Cu^2+^ stress.
2. Among all the known MTs from most organisms, the *Tetrahymena* MTs have several unique features. For example, they are considerably longer in size (≈67–74 % for CdMTs and ≈ 24-57 % for CuMTs), and therefore have higher molecular masses than standard MTs. CdMTs have relatively abundant CCC motifs with a strictly conserved modular-submodular structure and higher metal binding capacities.
3. Among the *Tetrahymena* CdMTs, the tri-modular structure seems to occur mainly in the *Borealis* group, while the bi-modular structure is found in the majority of species from the *Australis* group.
4. The remarkable asymmetry between *Tetrahymena* Cd- and CuMTs in codon usage for glutamine residues, corroborates the possibility that CuMTs could have diverged earlier than CdMTs in the *Tetrahymena* genus.
5. The gene expression patterns from the new *Tetrahymena* MT isoforms analyzed corroborate their multi-stress character. Likewise, a differential gene expression behavior among different MT isoforms is present in the same *Tetrahymena* species.

## Methods

### *Tetrahymena* species, culture conditions and stress treatments

The following five *Tetrahymena* species were used: *T. borealis* (SD 01609), *T. elliotti 4EA* (SD 01607), *T. americanis* (80C03), *T. patula LFF* (ATCC 50064), all supplied by ATCC (American Type Culture Collection) and *T. malaccensis 436*, supplied by the National *Tetrahymena* Stock Center (Cornell University). Cells were axenically grown in PP210 medium: 2 % v/v proteose peptone aqueous solution (Pronadisa) supplemented with 100 μM FeCl_3_ (Panreac), 200 μg/ml of streptomycin sulfate (Calbiochem) and 200 μg/ml of penicillin G (Sigma), maintained at a constant temperature (30 ± 1 °C).

Before RNA isolation, cell cultures were exposed to different stress conditions. Heavy-metal treatments were carried out for 1 or 24 h: 44.5 μM Cd^2+^ (CdCl_2_) (except for *T. borealis*, which was treated with 10 μM Cd^2+^), 315 μM Cu^2+^ (CuSO_4_ ∙ 5 H_2_O), 965 μM Pb^2+^ (Pb(NO_3_)_2_), 100 μM As^5+^ (Na_2_HAsO_4_) and 38.2 mM Zn^2+^ (ZnSO_4_ ∙ 7 H_2_O) (Sigma). Metal concentrations were approximately half the LC_50_ values calculated in *Tetrahymena thermophila* [72], resulting in insignificant cell mortality for each species used in this study, as it was checked by flow-cytometry (Additional file 2). Oxidative stress was induced by cell exposure (1 or 24 h) to 7.7 mM Paraquat (PQ) (Sigma) or 1 h to 2 mM Menadione (MD) (Sigma). As an apoptosis inducer, 100 μM Camptothecin (CAM) (Calbiochem) was used at 1 or 24 h exposures. Temperature stress was carried out by maintaining cells for 24 h in a cold (4 °C) or hot (42 °C) environment. pH stress was applied for 3 or 24 h under basic (pH 9) or acid (pH 5) conditions. All these treatments were carried out in PP210 growth medium. Finally, starvation conditions were induced using a buffer (0.01 mM TrisHCl pH 6.8) for 48 h or 4 days. Ultrapure reagent grade H_2_O was used in all experiments, with maximum conductivity of 18.2 MV obtained using a MILLI-Q water purification system (Millipore).

### Total DNA and RNA isolations and cDNA synthesis

Exponential cell cultures (1–3 × 10^5^ cells/ml) of the different *Tetrahymena* species were harvested by centrifugation at 2800 rpm for 3 min. Total DNA was isolated using the protocol described in [73] and samples were treated with 10 mg/ml RNase A (Thermo- Scientific) for 2 h at 37 °C. Total RNA samples were isolated from previously stressed exponential cell cultures using the TRIzol Reagent method (Invitrogen). RNA samples were treated with DNase I (Roche) for 30 min at 37 °C. DNA and RNA integrity was tested by agarose gel electrophoresis. MultiScribe Reverse Transcriptase 50 units/μl (Life Technologies) and oligo(dT)-adaptor primer (Roche) were used to synthesize the cDNAs from 3.5 μg of the total RNA samples.

### Standard PCR reactions, 5’/3’ RACE and cloning

Convergent degenerate primers (*MET1*/*MET2* and *MTCU1*/*MTCU2*) were designed, using the amino acid sequence of the CdMT TpyrMT-1 [14] and the nucleotide sequence of the CuMT *TtherMTT2* [19] in order to amplify putative CdMTs or CuMTs from different *Tetrahymena* species (Additional file 3). Standard PCR was carried out using the *AmpliTaq Gold PCR Master Mix* and 1.25 U/reaction of the *AmpliTaq Gold DNA Polymerase* (Applied Biosystems). The following PCR program was applied: 7 min at 94 °C, 30 cycles of 1 min at 94 °C, 1 min at 50 ± 3 °C and 2 min at 72 °C and finally, 5 min at 72 °C. The full-length of cDNA sequences was obtained using 3’ or 5’ RACE kits: *3’ RACE System for Rapid Amplification of cDNA Ends* (Invitrogen) or *5’ RACE System for Rapid Amplification of cDNA Ends* (Invitrogen). In the 3’ RACE system an adaptor primer (AP) and the AUAP primer supplied with the kit was generally used but in some cases we designed a specific primer for a specific sequence. AAP primer (Invitrogen) was used in the 5’ RACE, and we also designed a specific primer for each sequence (Additional file 3). PCR products were analyzed by standard 1.5 % agarose gel electrophoresis in TAE 1x buffer (40 mM Tris, 1 m MEDTA and 5.7 % glacial acetic acid) and stained with GelRed (Biotium) (3x in water). They were then cloned using the *TOPO TA Cloning Kit* (Invitrogen).

### Quantitative real-time RT-PCR

cDNA samples were amplified in duplicate in 96 microtiter plates (Applied Biosystems). Each PCR reaction (20 μl) contained: 10 μl of SBYR Green (Takara), 1 μl of each primer (at 300 nM final concentration), 3 μl of ultrapure sterile water and 5 μl of cDNA. PCR primers (Additional file 3) were designed using the “Primer Quest and Probe Design” online-application of IDT (*Integrated DNA Technologies*). α-tubulin and β-actin were used as the endogenous control or normalizer genes. Primer specificity was tested, melting curves were obtained and each PCR product was confirmed by gel electrophoresis and sequencing. Real-time RT-PCR reactions were carried out in an iQ5 real-time PCR apparatus (Bio-Rad). The thermal cycling protocol was as follows: 5 min at 95 °C, 40 cycles (30 s at 95 °C, 30 s at 55 °C and 20 s at 72 °C), 1 min at 95 °C and 1 min at 55 °C. All controls were negative (no template control or RT minus control). Amplification efficiency (E) was measured by using 10-fold serial dilutions of a positive control PCR template. The efficiency requirement was met for all the tested genes (Additional file 4). Results were finally processed by the standard-curve method (http://www6.appliedbiosystems.com/support/tutorials/pdf/performing_rq_gene_exp_rtpcr.pdf).

### DNA sequencing and *in silico* analysis of nucleotide and amino acid sequences

DNA sequences were obtained with the *ABI Prism 3730 DNA Analyser* sequencer (Applied Biosystem). In order to look for new MT sequences we also analyzed the macronuclear genomes of *T. malaccensis, T. borealis* and *T. elliotti* with the BLAST program on the *Tetrahymena Comparative Database* website (http://www.broadinstitute.org/scientific-comunity/science/programs/genome-sequencing-and-analysis/update-our-microbial-eukaryotes) and the macronuclear genome of *I. multifiliis* with the BLAST program on the *Ichthyophthirius Genome Database* website (http://ich.ciliate.org/index.php/home/welcome)*.* Multiple sequence alignments were performed using T-Coffee online application (which uses the Clustal Wallis method) (Tree-based Consistency Objective Function for Alignment Evaluation) [74]. The phylogenetic trees of both *Tetrahymena* MTs and SSrRNAs were built using the maximum likehood algorithm (MEGA 5.05, using the Poisson model). Phylogenetic trees (Newick) and alignments (FASTA) are available from TreeBase (http://purl.org/phylo/treebase/phylows/study/TB2:S18773, http://purl.org/phylo/treebase/phylows/study/TB2:S18776) with references: TB2:S18773 and TB2:S18776. Both, 5’ and 3’UTR conserved motifs were searched, based on our previous research work [19].

### Nucleotide sequence accession numbers

The 21 new cDNA sequences have been deposited in the GenBank Database. All GenBank accession numbers of MT genes referred on this article are shown in Additional file 5.

### Availability of data and materials

The datasets supporting the conclusions of this article are included within the article (and its Additional file 5) and are available in the TreeBase repository (http://purl.org/phylo/treebase/phylows/study) with references: TB2:S18773 and TB2:S18776.

## Additional files

Additional file 1: Theoretical metal binding capacity for *Tetrahymena* Cd- and CuMTs. (DOCX 16 kb) Additional file 2: Heavy metal toxicity analysis. (DOCX 17 kb) Additional file 3: Primers for standard PCR and qRT-PCR. (DOCX 16 kb) Additional file 4: Quantitative RT-PCR standard-curve parameters. (DOCX 18 kb) Additional file 5: Ciliate metallothionein GenBank accession numbers. (DOCX 17 kb)

## Abbreviations

CdMTs: cadmium-binding MTs
CuMTs: copper-binding-MTs
LC_50_: lethal concentration killing 50 % of cell population
MTs: metallothioneins
ORF: open reading frame
qRT-PCR: quantitative reverse transcription polymerase chain reaction
SSrRNA: small subunit ribosomal RNA
