# Supplementary material for: The Tetrahymena metallothionein gene family: twenty-one new cDNAs, molecular characterization, phylogenetic study and comparative analysis of the gene expression under different abiotic stressors
Source: BMC Genomics. 2016 May 10;17:346. doi: 10.1186/s12864-016-2658-6 (PMC4862169; doi:10.1186/s12864-016-2658-6)
Supplement: Additional file 1: — Theoretical metal binding capacity for Tetrahymena Cd- and CuMTs. (DOCX 16 kb) [file 12864_2016_2658_MOESM1_ESM.docx]

**Additional file 1**

**Theoretical metal binding capacity for *Tetrahymena* Cd- and CuMTs**

| **CdMTs** | **Theoretical binding capacity** | **CuMTs** | **Theoretical binding capacity** |
| --- | --- | --- | --- |
| TpigMT-1 | 12 | TpigMT-2 | 17 |
| TpyrMT-1 | 11 | TtheMTT2 | 19 |
| TpyrMT-2 | 19 | TtheMTT4 | 19 |
| TtheMTT1 | 17 | TrosMTT2 | 13 |
| TtheMTT3 | 15 | TtroMT1 | 17 |
| TtheMTT5 | 8 | TtroMTT2 | 19 |
| TrosMTT1 | 12 | Sp1.7-MT1 | 19 |
| TtroMTT1 | 16 | **TelliMTT6** | 26 |
| TmobMT1 | 18 | **TelliMTT8** | 16 |
| TvorMT1 | 17 | **TmalaMTT5** | 19 |
| ThegMT1 | 12 | **TborMTT3** | 26 |
| ThegMT2 | 17 | **TborMTT4** | 10 |
| ThegMT3 | 18 | **TborMTT6** | 26 |
| ThegMT4 | 18 | **TborMTT7** | 25 |
| **TborMTT1** | 12 | **TborMTT8** | 13 |
| **TborMTT2** | 17 | **TamerMTT3** | 19 |
| **TelliMTT1** | 16 | **ImMTT2** | 32 |
| **TelliMTT2** | 15 | Average | ≈ 20 |
| **TmalaMTT1** | 17 |  | |
| **TmalaMTT2** | 15 |  |  |
| **TmalaMTT3** | 16 |  |  |
| **TmalaMTT4** | 9 |  |  |
| **TpatMTT1** | 12 |  |  |
| **TpatMTT2** | 14 |  |  |
| **TamerMTT1** | 11 |  |  |
| **TamerMTT2** | 17 |  |  |
| Average | ≈ 15 |  |  |

New MTs are in bold text. See the text for further explanation.
