# Supplementary material for: The Tetrahymena metallothionein gene family: twenty-one new cDNAs, molecular characterization, phylogenetic study and comparative analysis of the gene expression under different abiotic stressors
Source: BMC Genomics. 2016 May 10;17:346. doi: 10.1186/s12864-016-2658-6 (PMC4862169; doi:10.1186/s12864-016-2658-6)
Supplement: Additional file 2: — Heavy metal toxicity analysis. (DOCX 17 kb) [file 12864_2016_2658_MOESM2_ESM.docx]

**Additional file 2**

**Heavy metal toxicity analysis carry out by flow-cytometry in four *Tetrahymena* species**

| **Heavy metal** | **Concentration (µM)** | **% mortality** | | | |
| --- | --- | --- | --- | --- | --- |
|  |  | *T. borealis* | *T. elliotti* | *T. americanis* | *T. malaccensis* |
| Cd^2+^ | 15 | 4.94 | 5.15 | 1.36 | 0.06 |
|  | 25 | 19.5 | 5.16 | 0.66 | 0.05 |
|  | **45** | 65.64* | 4.97 | 0.83 | 0.05 |
|  | 60 | 71.29 | 5.29 | 2.42 | 0.06 |
|  | 100 | 78.85 | 4.04 | 3.42 | 1.58 |
| Cu^2+^ | **300** | 0.44 | 2.37 | 2.83 | 0.12 |
|  | 500 | 1.75 | 1.14 | 1.81 | 0.13 |
|  | 1000 | 0.72 | 1.27 | 1.32 | 0.22 |
|  | 2000 | 1.09 | 1.57 | 1.73 | 0.6 |
|  | 4000 | 4.85 | 3.87 | 2.85 | 2.26 |
| Pb^2+^ | 500 | 1.44 | 2.21 | 1.31 | 0.05 |
|  | **1000** | 1.70 | 3.95 | 1.37 | 0.04 |
|  | 3000 | 2.57 | 1.21 | 3.61 | 0.14 |
|  | 5000 | 3.35 | 0.53 | 14.46 | 0.36 |
|  | 6000 | 4.08 | 0.81 | 36.23 | 1.53 |
| As^5+^ | **100** | 0.51 | 0.72 | 1.24 | 0.26 |
|  | 500 | 0.75 | 0.43 | 1.51 | 0.88 |
|  | 1000 | 0.91 | 0.35 | 1.21 | 1.46 |
|  | 3000 | 3.31 | 0.56 | 2.73 | 1.07 |
|  | 5000 | ND | 2.87 | 13.38 | 4.77 |
| Zn^2+^ | 1000 | 1.22 | 0.75 | 1.64 | 0.06 |
|  | **2000** | 13.47 | 3.42 | 6.22 | 0.94 |
|  | 2500 | 31.12 | 12.72 | 10.8 | 1.85 |
|  | 3000 | 26.91 | 26.68 | 15.69 | 2.53 |
|  | 5000 | 97.75 | 75.31 | 71.56 | 5.98 |

* We have not detected a significant cell mortality for all metal concentrations used to study the gene expression (numbers in bold). Excepting for *T. borealis*, that shown an excessive cell mortality (65.6%) at 45 μM (flow-cytometry analysis) or 44.5 μM (habitual concentration used in expression analysis). Therefore, we reduced the Cd^2+^ concentration to 10 µM to carry out the gene expression analysis in this *Tetrahymena* specie. ND: not determined.
