# Supplementary material for: The Tetrahymena metallothionein gene family: twenty-one new cDNAs, molecular characterization, phylogenetic study and comparative analysis of the gene expression under different abiotic stressors
Source: BMC Genomics. 2016 May 10;17:346. doi: 10.1186/s12864-016-2658-6 (PMC4862169; doi:10.1186/s12864-016-2658-6)
Supplement: Additional file 3: — Primers for standard PCR and qRT-PCR. (DOCX 16 kb) [file 12864_2016_2658_MOESM3_ESM.docx]

**Additional file 3**

**Primers for standard PCR and qRT-PCR**

| **Primer** | **Sequence (5´- 3´)** | **Use** |
| --- | --- | --- |
| MET 1^*^  MET 2^*^ | AAYTGYTGYTGYGGWGARAAY  TCRGTRCARCARGCYTTRGCY | Putative CdMT genes amplification |
| MTCU1^*^  MTCU2 | ATGGAYACTCAAACTTCAAAC  TTACAGTTGGAAGTAGAACC | Putative CuMT genes amplification |
| TborMTT1-  5RACE | CACCAGTACCTGTGCAGCAT | 5´RACE *TborMTT1* |
| TamerMTT1-  5RACE | CAGGTTTGCAGCATTAGCAA | 5´RACE *TamerMTT1* |
| TamerMTT3-  5RACE | TGCATTTGCAAGCAGAAGTC | 5´RACE *TamerMTT3* |
| 5RACE-  PAT1 | TTCCTCAGTGCAACAATCC | 5´RACE *TpatMTT1* |
| 5RACE-  PAT2 | CAAGAGCATCCACTGTTAGG | 5´RACE *TpatMTT2* |
| 3RACE-  ELLI2 | GCTGTAAACCTGAATAA | 3´RACE *TelliMTT2* |
| 3RACE-ELLI8 | GACTTCAGCTTGTAAAT | 3´RACE *TelliMTT8* |
| 3RACE-BOR8 | TTTCTGATGGTTGTAAG | 3´RACE *TborMTT8* |
| 3RACE-TMALA1 | TAGTGGATGCTGCTGTGTTAG | 3’RACE *TmalaMTT1* |
| 3RACE-TMALA2 | GGTTCTGCAGAGGGAAGTAAAT | 3´RACE *TmalaMTT2* |
| 3RACE-TMALA3 | GTGCTGCTGTCCTAGTGAAAT | 3´RACE *TmalaMTT3* |
| 3RACE-TMALA4 | TGAGTGTAAGTGTGGACCTAATG | 3´RACE *TmalaMTT4* |
| 3RACE-PAT2 | CCTAACAGTGGATGCTCTTG | 3´RACE *TpatMTT2* |
| ATUB1  ATUB2 | TGTCGTCCCCAAGGAT  GTTCTCTTGGTCTTGATGGT | qRT-PCR tubulin gene |
| TtACT1  TtACT2 | CTCTCTTTCTACCTTCCAAACT  AGGACCAGATTCATCATATTC | qRT-PCR actin gene |
| BOR1A  BOR1B | TGGATGTTGCTGTGTAAGTAA  TACAAGCATCACCAGTACCT | qRT-PCR *TborMTT1* |
| BOR2A  BOR2B | TGTACTGACTGTGAATGCTGTAA  CACCCACTCTTAGGATCTGTGC | qRT-PCR *TborMTT2* |
| BOR7A  BOR7B | TGTAGCTCCTGCTAGTGTAGT  AGAAGCAGAGACACCACATTT | qRT-PCR *TborMTT7* |
| ELLI6A  ELLI6B | GGAACTCACTCTGCTTCAACT  TCAGAGCATCCACAAGGATTA | qRT-PCR *TelliMTT6* |
| Tam-MTT3A  Tam-MTT3B | CCAACCTTGCGAAAACT  TTAGTGTGAGATCCACATT | qRT-PCR *TamerMTT3* |
| NTMALA5A  NTMALA5B | CGGATCCCACGCTGAAA  TGGAGGTAGAACCACACTTG | qRT-PCR *TmalaMTT5* |

Primers were synthesized by Invitrogen or IDT (Integrated DNA Technologies).

^*^ Degenerate primers: R = A or G; W = A or T; Y = C or T.
