# Supplementary material for: The Tetrahymena metallothionein gene family: twenty-one new cDNAs, molecular characterization, phylogenetic study and comparative analysis of the gene expression under different abiotic stressors
Source: BMC Genomics. 2016 May 10;17:346. doi: 10.1186/s12864-016-2658-6 (PMC4862169; doi:10.1186/s12864-016-2658-6)
Supplement: Additional file 4: — Quantitative RT-PCR standard-curve parameters. (DOCX 18 kb) [file 12864_2016_2658_MOESM4_ESM.docx]

**Additional file 4**

**Quantitative RT-PCR standard-curve parameters for each MT and expression control genes**

| **Species** | **Gene** | **Slope** | **Efficiency (%)** | **y-intercept** | **R^2 (*)^** |
| --- | --- | --- | --- | --- | --- |
| *T. borealis* | *α-tubulin* | -3.644 | 88.10 | 14.044 | 0.997 |
|  | *β-actin* | -3.180 | 106.30 | 22.256 | 0.999 |
|  | *TborMTT1* | -3.462 | 94.47 | 17.989 | 0.996 |
|  | *TborMTT2* | -3.375 | 97.83 | 17.738 | 0.990 |
|  | *TborMTT7* | -3.271 | 102.20 | 16.265 | 0.991 |
| *T. elliotti* | *α-tubulin* | -3.494 | 93.90 | 11.692 | 0.975 |
|  | *β-actin* | -3.668 | 87.40 | 10.024 | 0.989 |
|  | *TelliMTT6* | -3.271 | 102.20 | 15.800 | 0.994 |
| *T. americanis* | *α-tubulin* | -3.212 | 104.80 | 10.713 | 0.993 |
|  | *β-actin* | -3.474 | 94.00 | 15.072 | 0.997 |
|  | *TamerMTT3* | -3.436 | 95.40 | 17.184 | 0.994 |
| *T. malaccensis* | *α-tubulin* | -3.459 | 94.60 | 12.450 | 0.962 |
|  | *β-actin* | -3.565 | 90.80 | 18.589 | 0.986 |
|  | *TmalaMTT5* | -3.577 | 90.40 | 18.876 | 0.982 |

*correlation coefficient.
