# Supplementary material for: The Tetrahymena metallothionein gene family: twenty-one new cDNAs, molecular characterization, phylogenetic study and comparative analysis of the gene expression under different abiotic stressors
Source: BMC Genomics. 2016 May 10;17:346. doi: 10.1186/s12864-016-2658-6 (PMC4862169; doi:10.1186/s12864-016-2658-6)
Supplement: Additional file 5: — Ciliate metallothionein GenBank accession numbers. (DOCX 17 kb) [file 12864_2016_2658_MOESM5_ESM.docx]

**Additional file 5**

**Ciliate metallothionein (MT) GenBank accession numbers**

| **Gene** | **Specie** | **Accession number** |
| --- | --- | --- |
| *TpigMT-1* | *Tetrahymena pigmentosa* | EU420056 |
| *TpigMT-2* | *Tetrahymena pigmentosa* | AF479586 |
| *TpyrMT-1* | *Tetrahymena pyriformis* | AJ005080 |
| *TpyrMT-2* | *Tetrahymena pyriformis* | AY765220 |
| *TtheMTT1* | *Tetrahymena thermophila* | AY061892 |
| *TtheMTT2* | *Tetrahymena thermophila* | AY204351 |
| *TtheMTT3* | *Tetrahymena thermophila* | AY740525 |
| *TtheMTT4* | *Tetrahymena thermophila* | AY660008 |
| *TtheMTT5* | *Tetrahymena thermophila* | DQ517936 |
| *TrosMTT1* | *Tetrahymena rostrata* | EU627174 |
| *TrosMTT2* | *Tetrahymena rostrata* | EU627175 |
| *TtroMTT1* | *Tetrahymena tropicalis* | ABM74559 |
| *TtropMT1* | *Tetrahymena tropicalis* | FJ664125 |
| *TtropMT2* | *Tetrahymena tropicalis* | FJ664126 |
| *TmobMT1* | *Tetrahymena mobilis* | HQ166888 |
| *TvorMT1* | *Tetrahymena vorax* | HQ166889 |
| *ThegMT1* | *Tetrahymena hegewischi* | HQ166890 |
| *ThegMT2* | *Tetrahymena hegewischi* | HQ166891 |
| *ThegMT3* | *Tetrahymena hegewischi* | HQ166892 |
| *ThegMT4* | *Tetrahymena hegewischi* | HQ166893 |
| *Sp1.7-MT1* | *Tetrahymena sp 1.7* | HE820725 |
| ***TborMTT1*** | *Tetrahymena borealis* | KU052675 |
| ***TborMTT2*** | *Tetrahymena borealis* | KU052676 |
| ***TborMTT3*** | *Tetrahymena borealis* | KU052677 |
| ***TborMTT4*** | *Tetrahymena borealis* | KU167650 |
| ***TborMTT6*** | *Tetrahymena borealis* | KU052678 |
| ***TborMTT7*** | *Tetrahymena borealis* | KU052679 |
| ***TborMTT8*** | *Tetrahymena borealis* | KU052680 |
| ***TelliMTT1*** | *Tetrahymena elliotti* | KU167642 |
| ***TelliMTT2*** | *Tetrahymena elliotti* | KU167643 |
| ***TelliMTT6*** | *Tetrahymena elliotti* | KU167644 |
| ***TelliMTT8*** | *Tetrahymena elliotti* | KU167645 |
| ***TmalaMTT1*** | *Tetrahymena malaccensis* | KU167646 |
| ***TmalaMTT2*** | *Tetrahymena malaccensis* | KU167647 |
| ***TmalaMTT3*** | *Tetrahymena malaccensis* | KU167648 |
| ***TmalaMTT4*** | *Tetrahymena malaccensis* | KU167649 |
| ***TmalaMTT5*** | *Tetrahymena malaccensis* | KU167651 |
| ***TpatMTT1*** | *Tetrahymena patula* | KU167652 |
| ***TpatMTT2*** | *Tetrahymena patula* | KU167653 |
| ***TamerMTT1*** | *Tetrahymena americanis* | KU052681 |
| ***TamerMTT2*** | *Tetrahymena americanis* | KU167641 |
| ***TamerMTT3*** | *Tetrahymena americanis* | KU167655 |
| ***ImMTT2*** | *Ichthyophthirius multifiliis* | KU167654 |

The 22 new metallothionein genes reported in this paper are

in bold text.
